# Chasing the Critical Wetting Transition. An Effective Interface Potential Method

**DOI:** 10.3390/ma14237138

**Published:** 2021-11-24

**Authors:** Paweł Bryk, Artur P. Terzyk

**Affiliations:** 1 Faculty of Chemistry, Maria Curie Skłodowska University, 20-031 Lublin, Poland; bryk@hektor.umcs.lublin.pl; 2 Physicochemistry of Carbon Materials Research Group, Faculty of Chemistry, Nicolaus Copernicus University, 87-100 Toruń, Poland

**Keywords:** wetting, computer simulation, Ising model

## Abstract

Wettablity is one of the important characteristics defining a given surface. Here we show that the effective interface potential method of determining the wetting temperature, originally proposed by MacDowell and Müller for the surfaces exhibiting the first order wetting transition, can also be used to estimate the wetting temperature of the second order (continuous) wetting transition. Some selected other methods of determination of the wetting temperature are also discussed.

## 1. Introduction

Understanding and controlling wetting properties of materials are some of the most important factors in many industrial applications including oil recovery [1], mineral flotation [2] and design of superamphiphobic surfaces [3].

Wetting transition is a surface-induced transition in which the contact angle of a liquid deposited on a surface drops to zero from a non-zero value upon increasing temperature. This transition has been the subject of many theoretical and experimental studies [4,5,6,7,8,9,10,11,12]. The wetting transition can be either first order or continuous (second-order). In the case of the first-order wetting transition there is additional prewetting (thin-thick film) transition, which occurs off-coexistence and the prewetting line joins the binodal exactly at the wetting point. The critical (second-order) wetting transition is not accompanied by prewetting.

The nature of the wetting transition and its universality class depends on (among others) the dimensionality of the system and the range of interparticle interactions. While in most cases the nature of the wetting transition has been well established and documented, it turns out that the case of 3-dimensional critical wetting transition for short-ranged forces (i.e., decaying exponentially, or faster) posed significant problems.

One of the important early discoveries was that the critical wetting transition for short-range forces is nonuniversal. Using renormalization-group calculations based on an effective interfacial Hamiltonian Brezin et al. [8] predicted that the critical exponent for this transition depends on a dimensionless parameter

(1) $$\omega =\frac{k_{B}T}{4\pi \Sigma \xi^{2}}$$

where *T* is the temperature, ξ is the correlation length of the bulk wetting phase and Σ is the interfacial stiffness. Capillary-wave-like fluctuations give rise to a diverging transverse correlation length $\xi_{∥}$ and the relevant exponent is believed to behave

(2) $$\nu_{∥}=\left\{\begin{array}{cc} {\left(1-\omega \right)}^{-1} & \;\;\;\mathrm{for}\;\;\;0<\omega <1/2\\ {\left(\sqrt{2}-\sqrt{\omega }\right)}^{-2} & \;\;\;\mathrm{for}\;\;\;1/2<\omega <2\\ \infty & \;\;\;\mathrm{for}\;\;\;\omega >2\;. \end{array}\right.$$

Unfortunately, subsequent Monte Carlo calculations carried out for the Ising model [13,14,15] revealed that while the general features regarding the wetting transition for the Ising model do agree with theoretical predictions [9], the critical wetting transition is only very weakly nonuniversal. This disagreement has been the subject of ever-lasting efforts in order to bridge the gap between the theory and simulations. Using nonlocal effective interfacial Hamiltonian Parry et al. [16] argued that the spectrum of the interfacial fluctuations has a lower cutoff due to appearance of a new length scale $\xi_{NL}=\sqrt{l\xi }\propto \sqrt{\ln\xi_{∥}}$. This gives rise to an effective wetting parameter, $\omega_{eff}$, of the form

(3) $$\omega_{eff}=\omega -\sqrt{2\omega^{3}}\frac{\ln(\kappa l)}{\kappa l}$$

where $\kappa =\xi^{-1}$, and *l* denotes film thickness. This leads to lowering of the value of the effective wetting parameter and yields lower effective critical exponent.

On the simulational front Albano and Binder [17] suggested that anisotropic finite size scaling (AFSS) theory should be suitable for studying wetting transitions. Using this approach Bryk and Binder [18] were able to recalculate the location of the wetting transition and confirm non-mean field character of critical wetting in 3D.

The main obstacle that hampered progress in our understanding of the nature of the critical wetting transition could be traced back to difficulties in accurate locating the critical wetting point from simulation. In the present work we discuss three methods of locating critical wetting from simulations. We show that the effective interface method can be used for locating the critical wetting transition. Two other alternative methods are also discussed.

## 2. Materials and Methods

Our model consists of cubic lattice of dimensions L×L×D with two free boundary layers L×L located at z=1 and z=D, and periodic boundary conditions in the remaining directions. The pseudospin variable at a lattice site *i* takes values $s_{i}=\pm 1$. The Hamiltonian for the system is

(4) $$H=-J\sum_{bulk}s_{i}s_{j}-J_{s}\sum_{surf}s_{i}s_{k}-H\sum_{bulk}s_{i}-H_{1}\sum_{k\in z=1}s_{k}-H_{D}\sum_{k\in z=D}s_{k}\;.$$

In the two free surface layers the exchange constant is $J_{s}$, otherwise the exchange constant is *J* throughout. The bulk field is *H*, and the surface fields acting on the first and last layer are $H_{1}$ and $H_{D}$, respectively. We consider three different types of systems, namely the symmetric systems with $H_{1}=H_{D}$, non-symmetric systems with $H_{1}\neq H_{D}$, $H_{D}=0$, and the anti-symmetric systems with $H_{1}=-H_{D}$. Throughout this study we restrict ourselves to the case $J_{s}=J$.

The systems were simulated using fast multispin coding algorithm [19]. In order to achieve better statistics we used the preferential sampling technique, so that, on average, 9 out of 10 samplings occurred in the region of interest (in the vicinity of the walls).

When simulating systems close to the critical wetting point the so-called critical slowing down hampers the statistics of the accumulated data. In order to overcome this drawback we applied hyper-parallel tempering technique [20] and simulated many systems at the same time. The swaps of spins between the systems *m* and *n* were accepted with the probability

(5) $$P_{nm}=\min\left[1,\exp\left(-\Delta \beta \Delta E\right)-\Delta \left(\beta H_{1}\right)\Delta m_{1}-\Delta \left(\beta H_{D}\right)\Delta m_{D}-\Delta \left(\beta H\right)\Delta m\right]$$

where

(6) $$\Delta \beta =\frac{1}{k_{B}T_{n}}-\frac{1}{k_{B}T_{m}}\;\;,\;\;\Delta E=E_{n}-E_{m}\;\;$$

(7) $$\Delta \left(\beta H_{1}\right)=\frac{1}{k_{B}T_{n}}H_{1}^{\left(n\right)}-\frac{1}{k_{B}T_{m}}H_{1}^{\left(m\right)}\;\;,\;\;\Delta m_{1}=m_{1}^{\left(n\right)}-m_{1}^{\left(m\right)}\;\;$$

(8) $$\Delta \left(\beta H_{D}\right)=\frac{1}{k_{B}T_{n}}H_{D}^{\left(n\right)}-\frac{1}{k_{B}T_{m}}H_{D}^{\left(m\right)}\;\;,\;\;\Delta m_{D}=m_{D}^{\left(n\right)}-m_{D}^{\left(m\right)}\;\;$$

(9) $$\Delta \left(\beta H\right)=\frac{1}{k_{B}T_{n}}H^{\left(n\right)}-\frac{1}{k_{B}T_{m}}H^{\left(m\right)}\;\;,\;\;\Delta m=m^{\left(n\right)}-m^{\left(m\right)}\;\;.$$

In the above $E_{n}$ and $T_{n}$ are the energy and the temperature of the system *n*. $k_{B}$ is the Boltzmann constant. Among other quantities of interest accumulated during a simulation were the magnetization in the surface layer

(10) $$m_{1}={\left(2L^{2}\right)}^{-1}\sum_{k\in surf1}<s_{k}>\;,$$

total magnetization

(11) $$m={\left(L^{2}D\right)}^{-1}\sum_{i}<s_{i}>\;,$$

and “mixed” surface layer susceptibility

(12) $$\chi_{1}=\frac{\partial m_{1}}{\partial H}=L^{2}D\left(<m_{1}m>-<m_{1}><m>\right)/k_{B}T\;.$$

## 3. The Effective Interface Potential Method

Tracking down the critical wetting transition point from computer simulation proved to be a challenge. MacDowell and Müller (MM), proposed a method [21,22] (hereinafter referred to as the effective interface potential (EIP) method) that relies on determining the distribution probability of magnetization from which the effective interface potential can be determined. We implemented this method with slight modifications. The key quantity is the probability $P(m_{p})$ of finding a system with magnetization $m_{p}$ defined as

(13) $$m_{p}={\left(D_{r}L^{2}\right)}^{-1}\sum_{k=1}^{D_{r}}<s_{k}>\;,$$

where $D_{r}$ denotes the range of interest, $D/2\leq D_{r}\leq D$. We considered the non-symmetric system with $H_{D}=0$. Following [21,22] we split the calculations of the order parameter distribution in windows and applied the successive sampling technique but with windows on $m_{p}$ rather than on *m*. Since we used hyper-parallel tempering two or three windows were deemed sufficient. From the order parameter distribution we obtained the effective interaction potential (up to a constant) via

(14) $$V_{eff}\left(m_{p}\right)/k_{B}T=-ln\left(P\left(m_{p}\right)\right)\;.$$

In our work, we used $P(m_{p})$, while MacDowell and Müller [21,22] calculated the adsorption distribution P(Γ). However the two distributions are closely related as one can recover the adsorption from $m_{p}$ by subtracting bulk magnetization.

It is important to note that the effective interface potential employed by us $V_{eff}\left(m_{p}\right)$ differs from that used traditionally in the literature [4,5,8]. Those authors considered $V_{eff}\left(l\right)$ where *l* is a distance of the interface from the wall, a local quantity. $V_{eff}\left(m_{p}\right)$ makes use of $m_{p}$—a global measure which translates into the mean distance from the wall only within mean field description. In other words our effective interface potential approach includes all the fluctuation effects, like interface overhangs and droplet excitations, which are not included in the local description. Since our interface potential carries a bulk term it cannot be directly used as an interface potential in conventional interface Hamiltonians. In such a case one can use a method proposed in [23].

MacDowell and Müller (and others [24,25,26,27,28,29,30]) have applied the outlined above approach to the long-range substrate potentials exhibiting the first-order wetting transition, where one expects the mean-field effective interface potential to be sufficient. It is natural to ask whether this approach works at all beyond mean-field. In order to answer this question we first carried out MC calculations for the 2D Ising model with short-range boundary fields. It is well established that for short-range forces in 2D wetting is completely dominated by the fluctuation effects [4]. An advantage of this procedure is that the simulational results can be compared with the exact solution for the wetting ordering field by Abraham [7]

(15) $$\exp\left(2J/k_{B}T\right)\left[\cosh\left(2J/k_{B}T\right)-\cosh\left(2H_{1c}/k_{B}T\right)\right]=\sinh\left(2J/k_{B}T\right)\;.$$

These calculations provide a clear-cut, stringent test of the proposed approach.

**Figure 1:**
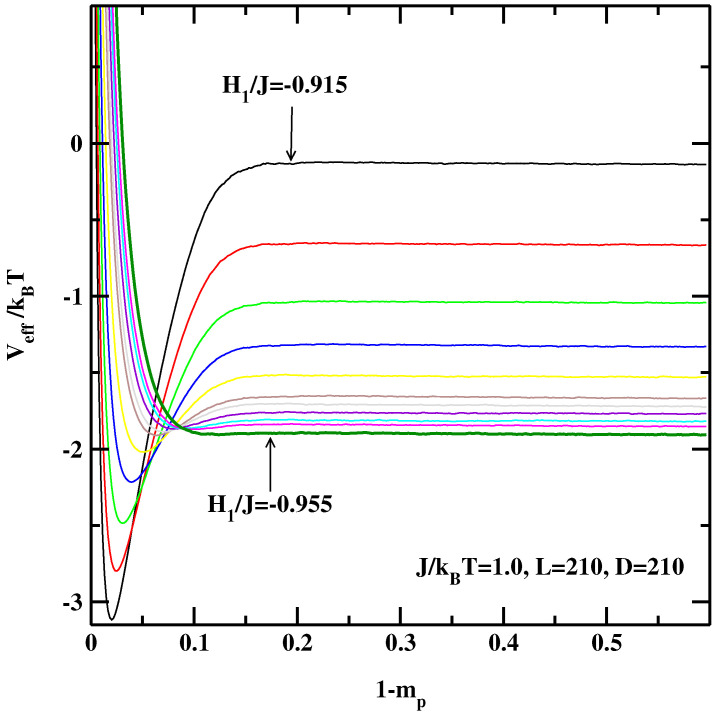
The effective interface potentials calculated for the 2D non-symmetric system at $J/k_{B}T=1.0$ and for L=210,D=210. The potentials are calculated for the surface fields $H_{1}/J=-0.915$, −0.92, −0.925, −0.93, −0.935, −0.94, −0.942, −0.945, −0.948, −0.950, and −0.955. The thick line corresponding to $H_{1}/J=-0.955$ denotes the effective interface potential with no detectable local minimum.

**Figure 2:**
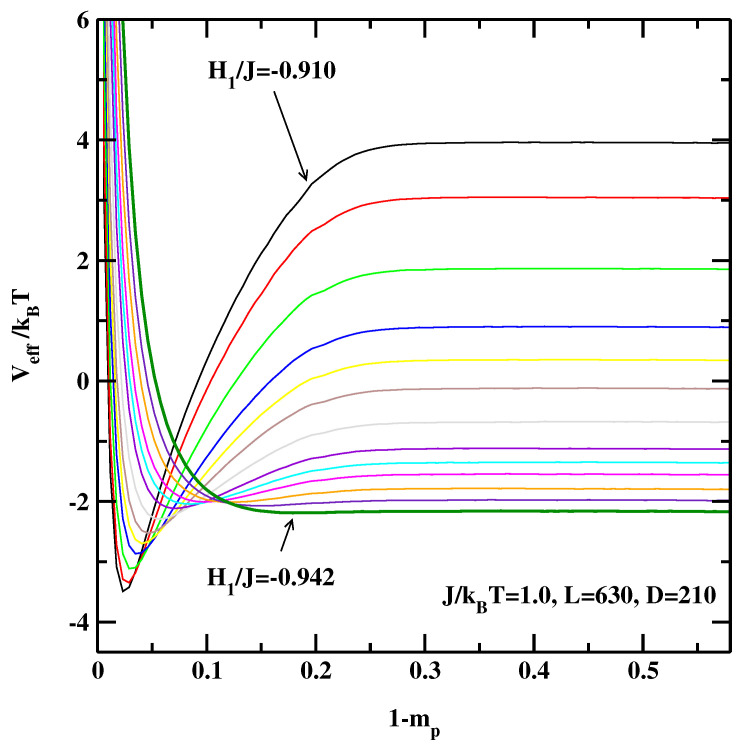
The effective interface potentials calculated for the 2D non-symmetric system at $J/k_{B}T=1.0$ and for L=630,D=210. The potentials are calculated for the surface fields $H_{1}/J=-0.910$, −0.912, −0.915, −0.918, −0.920, −0.922, −0.925, −0.928, −0.930, −0.932, −0.935, −0.938, and −0.942. The thick line corresponding to $H_{1}/J=-0.942$ denotes the effective interface potential with no detectable local minimum.

**Figure 3:**
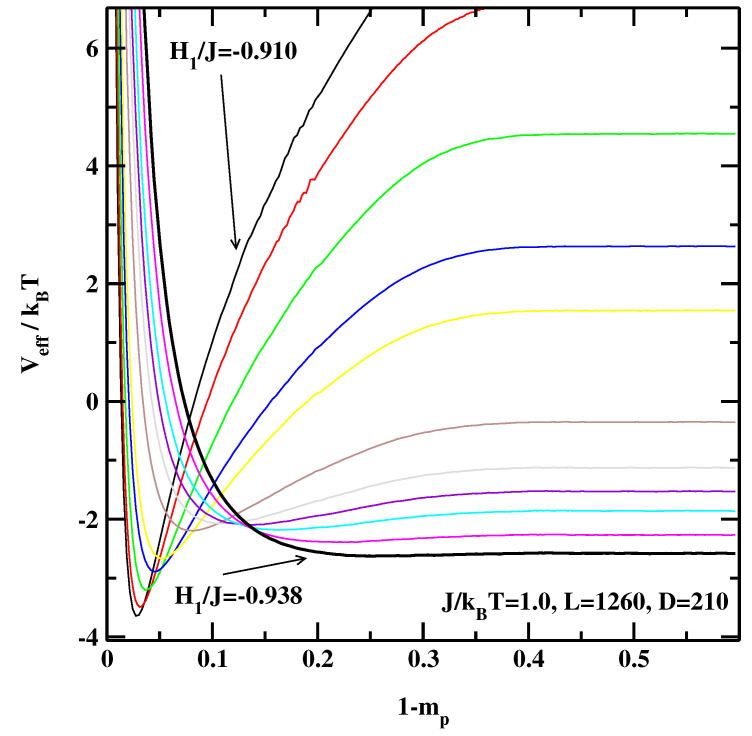
The effective interface potentials calculated for the 2D non-symmetric system at $J/k_{B}T=1.0$ and for L=1260,D=210. The potentials are calculated for the surface fields $H_{1}/J=-0.910$, −0.912, −0.915, −0.918, −0.920, −0.925, −0.928, −0.930, −0.932, −0.935, and −0.938. The thick line corresponding to $H_{1}/J=-0.938$ denotes the effective interface potential with no detectable local minimum.

Figure 1, Figure 2 and Figure 3 show the EIPs calculated for three system sizes, L=210, 630 and 1260 at $J/k_{B}T=1.0$. For smaller absolute values of the surface fields we observe a deep local minimum indicating that the system is in the partial wetting state. For these state points the successive sampling technique is particularly useful as it would be very difficult to obtain smooth effective interface potentials only from one simulation. As $H_{1}$ becomes more negative this minimum gradually becomes shallower and finally disappears completely indicating the critical wetting point for a system with the size *L*.

**Figure 4:**
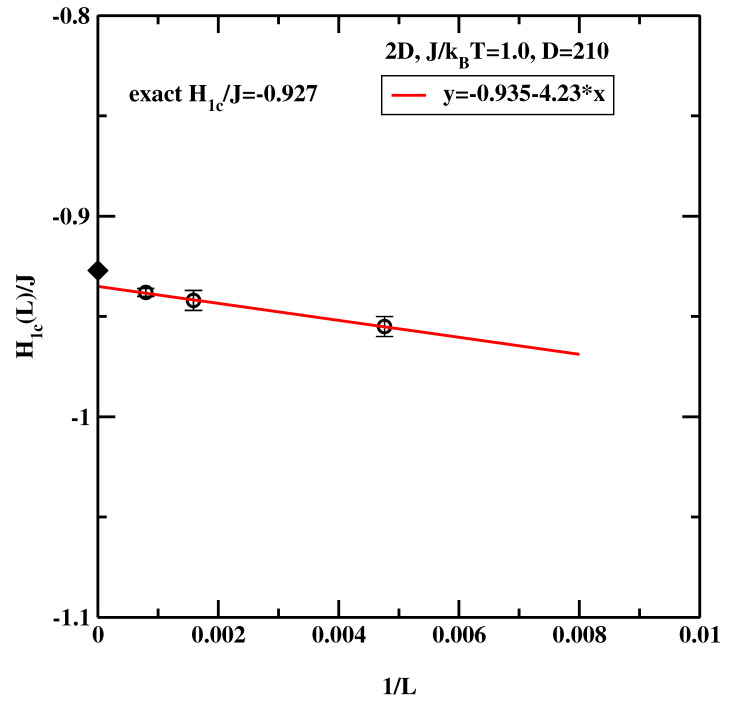
The estimate of the wetting surface field for the 2D system at $J/k_{B}T=1.0$ Circles correspond to the surface critical fields obtained from simulations. The straight line denotes a regression fit. The black diamond denotes the exact result [7].

Figure 4 shows extrapolation of the critical surface field to the thermodynamic limit L→∞. We note, that the simulational value of $H_{1c}\left(\infty \right)/J=-0.935$ agrees very well with the exact result, −0.927. Another important observation is that the critical surface field $H_{1c}\left(L\right)$ depends quite visibly on *L*, indicating that finite size corrections are important. This is in line with theoretical predictions of 2D critical wetting [4], i.e., the interfacial fluctuations in 2D renormalize the temperature (or alternatively the critical surface field) of the critical wetting transition.

**Figure 5:**
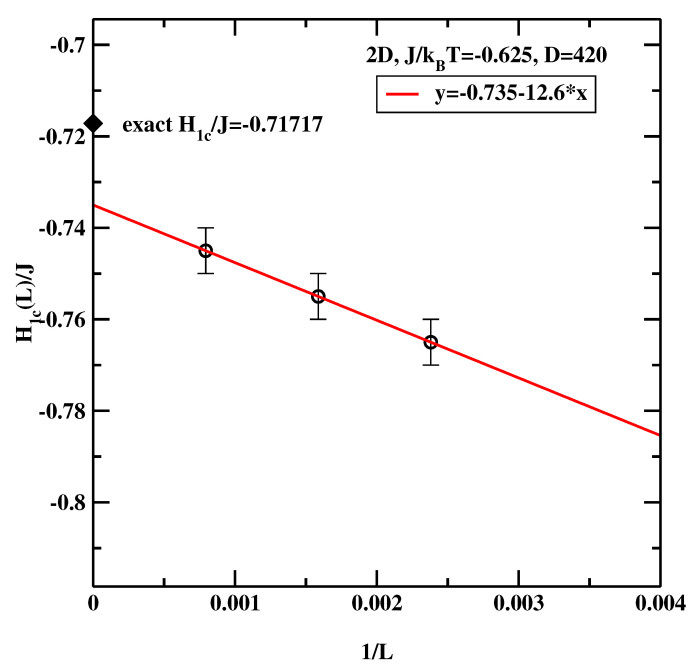
The estimate of the wetting surface field for the 2D system at $J/k_{B}T=0.625$ Circles correspond to the surface critical fields obtained from simulations. The straight line denotes a regression fit. The black diamond denotes the exact result [7].

It is instructive to see how accurate is the EIP method at higher temperatures (recall, that the inverse bulk critical temperature $J/k_{B}T_{c}=ln\left(1+\sqrt{2}\right)/2\approx 0.440687$). Figure 5 shows extrapolation of the critical surface field to the thermodynamic limit at $J/k_{B}T=0.625$ (the relevant effective potentials are not shown, for the sake of brevity). We observe that the simulational result $H_{1c}/J=-0.735$ now differs from the exact result (−0.71717) by almost 2.5%. This deterioration in accuracy can be traced back to the fact that in 2D (unlike in 3D) bulk fluctuations persist down to quite low temperatures. Since these fluctuations lead to broadening of $P(m_{p})$ it is inevitable that the method becomes less robust as the temperature gets closer to the bulk critical temperature. Hence, one important restriction of the EIP method is that it is applicable to systems with well separated bulk and interfacial fluctuations.

**Figure 6:**
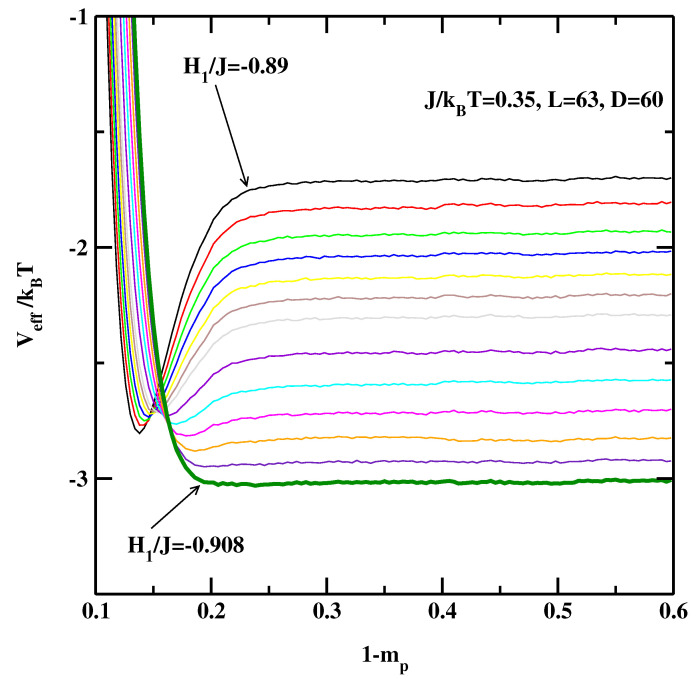
The effective interface potentials calculated for the 3D non-symmetric system at $J/k_{B}T=0.35$ and for L=63,D=60. The potentials are calculated for surface fields from $H_{1}/J=-0.89$, −0.891, −0.892, −0.893, −0.894, −0.895, −0.896, −0.898, −0.90, −0.902, −0.904, −0.906, and −0.908. The thick line corresponding to $H_{1}/J=-0.908$ denotes the effective interface potential with no detectable local minimum.

**Figure 7:**
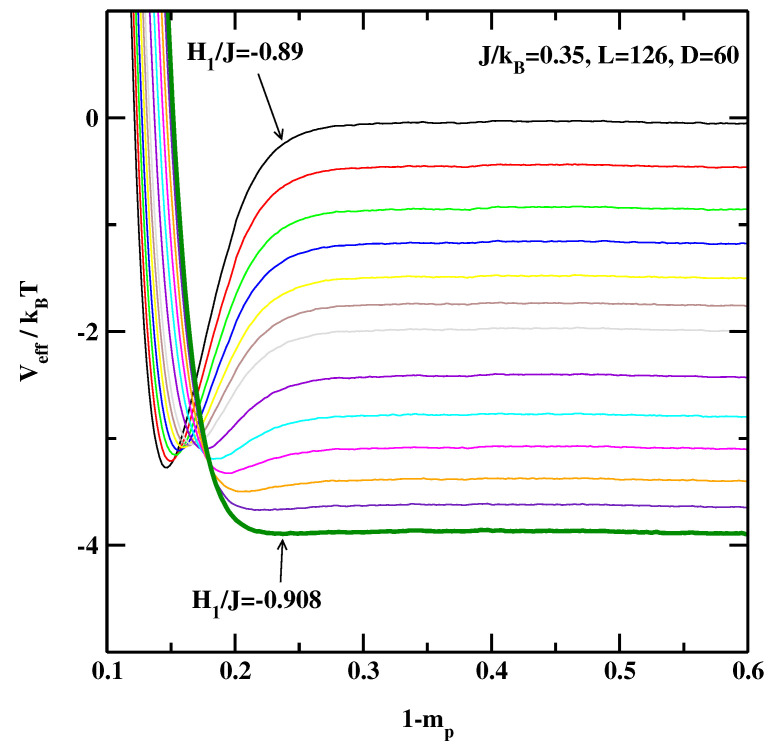
The effective interface potentials calculated for the 3D non-symmetric system at $J/k_{B}T=0.35$ and for L=126,D=60. The potentials are calculated for surface fields $H_{1}/J=-0.89$, −0.891, −0.892, −0.893, −0.894, −0.895, −0.896, −0.898, −0.90, −0.902, −0.904, −0.906, and −0.908. The thick line corresponding to $H_{1}/J=-0.908$ denotes the effective interface potential with no detectable local minimum.

**Figure 8:**
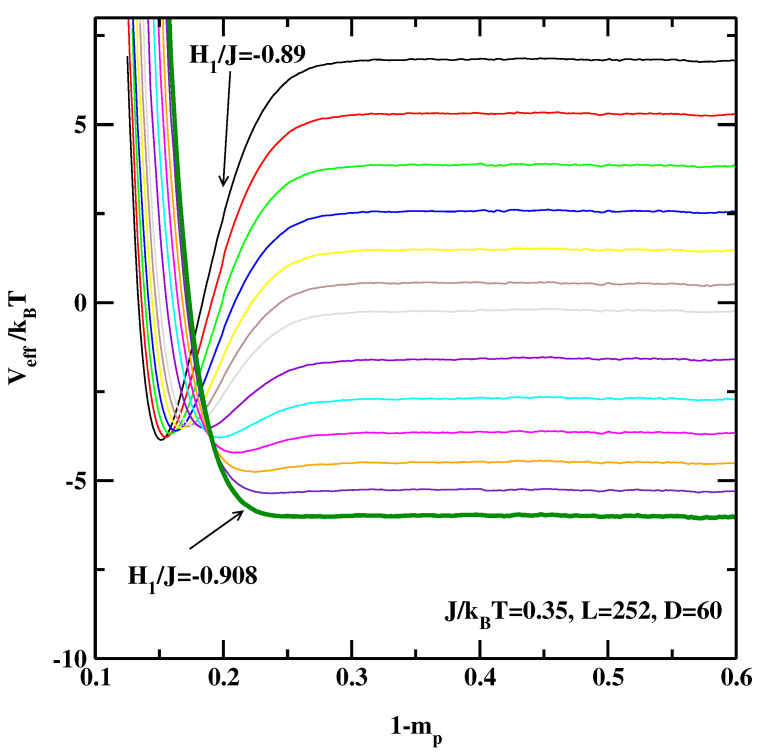
The effective interface potentials calculated for the 3D non-symmetric system at $J/k_{B}T=0.35$ and for L=252,D=60. The potentials are calculated for surface fields $H_{1}/J=-0.89$, −0.891, −0.892, −0.893, −0.894, −0.895, −0.896, −0.898, −0.90, −0.902, −0.904, −0.906, and −0.908. The thick line corresponding to $H_{1}/J=-0.908$ denotes the effective interface potential with no detectable local minimum.

Let us turn to the 3D Ising systems. Figure 6, Figure 7 and Figure 8 show the effective interface potentials calculated for $J/k_{B}T=0.35$, D=60, and for L=63, 126, and 252. We notice that even for the smallest system, L=63 (cf. Figure 6), the system with the surface field $H_{1}/J=-0.89$ is still in the partial wetting regime as there is a well visible minimum (as a side comment, this value was quoted as the critical surface field in Refs. [13,14,15]). We estimate that the wetting transition is located at $H_{1}/J=-0.908\pm 0.002$. Interestingly, for the larger systems (cf. Figure 7 and Figure 8) the transition is located also at $H_{1}/J=-0.908\pm 0.002$, i.e., there are no discernible finite-size effects.

**Figure 9:**
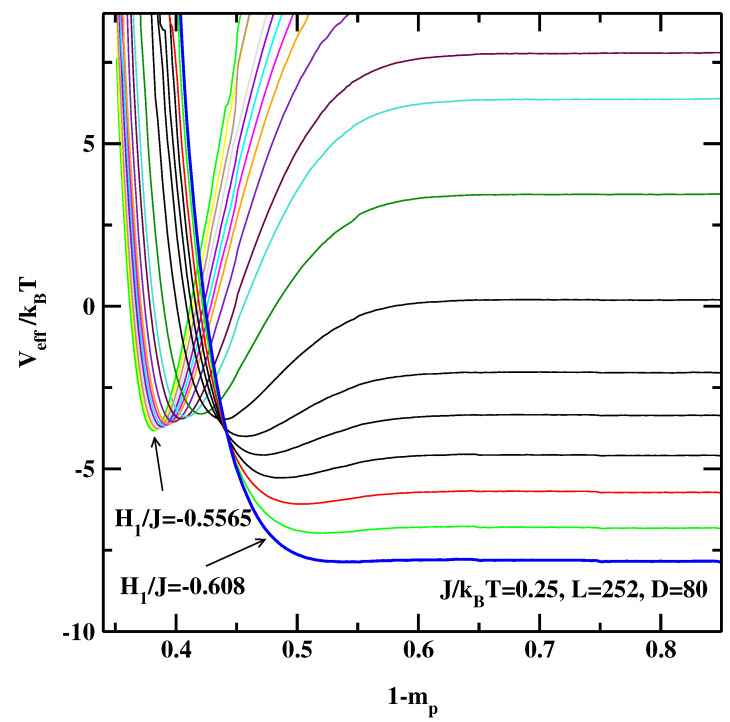
The effective interface potentials calculated for the non-symmetric system at $J/k_{B}T=0.25$ and for L=252,D=80. The potentials are calculated for surface fields from top $H_{1}/J=-0.5565$, −0.557, −0.558, −0.559, −0.560, −0.561, −0.562, −0.563, −0.564, −0.565, −0.568, −0.570, −0.575, −0.582, −0.588, −0.592, −0.596, −0.600, −0.604, and −0.608. The thick line corresponding to $H_{1}/J=-0.608$ denotes the effective interface potential with no detectable local minimum.

We find similar behaviour of the EIPs for $J/k_{B}T=0.25$. Independent of the system size we estimate the critical surface field $H_{1c}/J=-0.608\pm 0.004$ (the EIPs for the largest considered system size, L=256 are presented in. Figure 9). This is again in line with our knowledge about the critical wetting transition, i.e., in 3D the interfacial fluctuations do not renormalize the temperature of the wetting transition [8].

## 4. Other Methods of Determination of the Critical Surface Field

### 4.1. Determination of the Critical Surface Field by Thermodynamic Integration

A new method for determination of the contact angle has been proposed in Refs. [31,32]. This technique can be interpreted as a variant of the thermodynamic integration method (TIN), whereby a series of calculations is carried out for anti-symmetric fields, $H_{1}<0$, $H_{D}=-H_{1}$, starting from $H_{1}=0$. The contact angle can be determined from the relation

(16) $$\cos\left(\theta \right)=-\int_{0}^{H_{D}}\frac{m_{D}+m_{1}}{\gamma_{lv}}dH_{D}^{\prime }\;,$$

where $\gamma_{lv}$ is the surface tension [33,34]. The advantage of this approach is that the system that has to be simulated does not undergo a critical wetting transition. Consequently there are no diverging length scales and no critical fluctuations. The downside is that for critical wetting the contact angle goes to 0 tangentially,

(17) $$1-\cos\left(\theta \right)∼t^{2-\alpha_{s}}\;,$$

where $\alpha_{s}$ is the surface critical exponent for specific heat, $t=\frac{H_{1c}-H_{D}}{H_{1c}}$. This makes the finite-size analysis a delicate issue.

**Figure 10:**
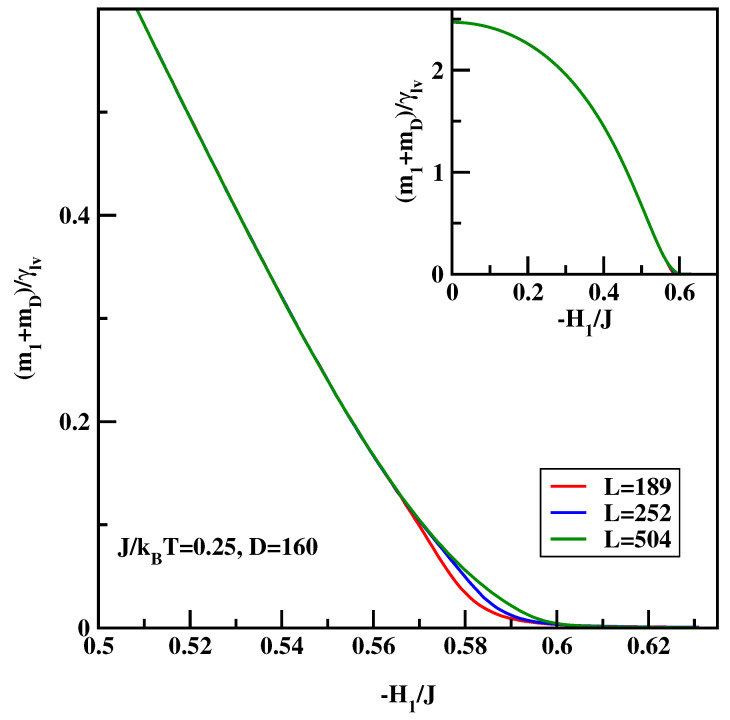
The integrand of Equation (16) vs. the surface field for $J/k_{B}T=0.25$ and for three system sizes *L* listed in Figure. The inset shows zoom-out of the main Figure.

**Figure 11:**
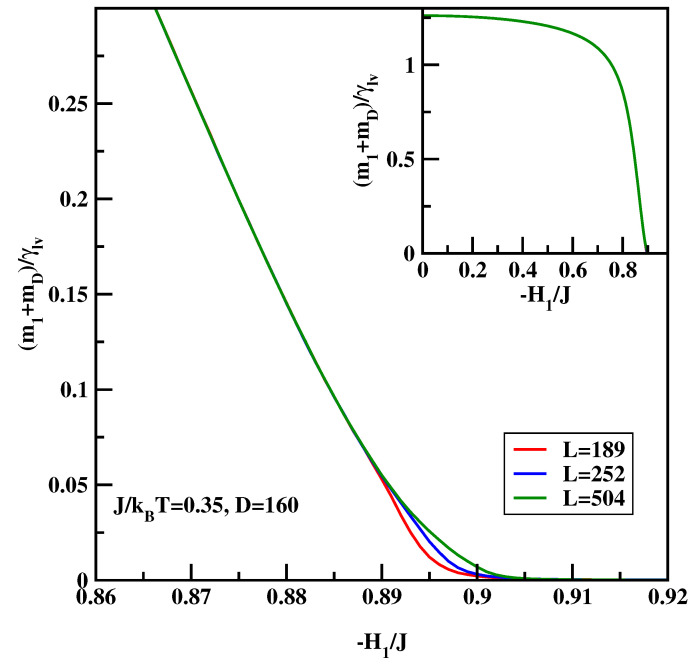
The integrand of Equation (16) vs. the surface field for $J/k_{B}T=0.35$ and for three system sizes *L* listed in Figure. The inset shows zoom-out of the main Figure.

**Figure 12:**
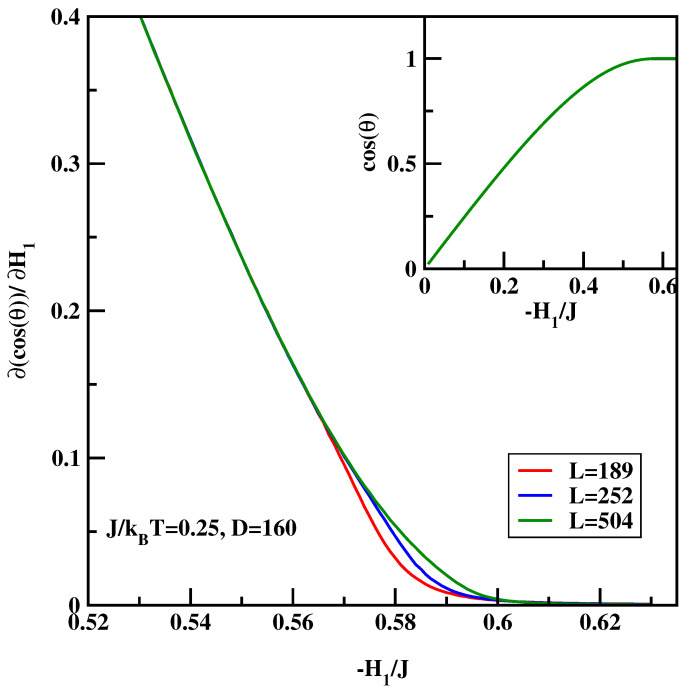
$\partial \cos\left(\theta \right)/\partial H_{1}$ vs. the surface field for $J/k_{B}T=0.25$ and for three system sizes *L* listed in Figure. The inset shows full dependence of cos(θ) vs. $H_{1}/J$.

**Figure 13:**
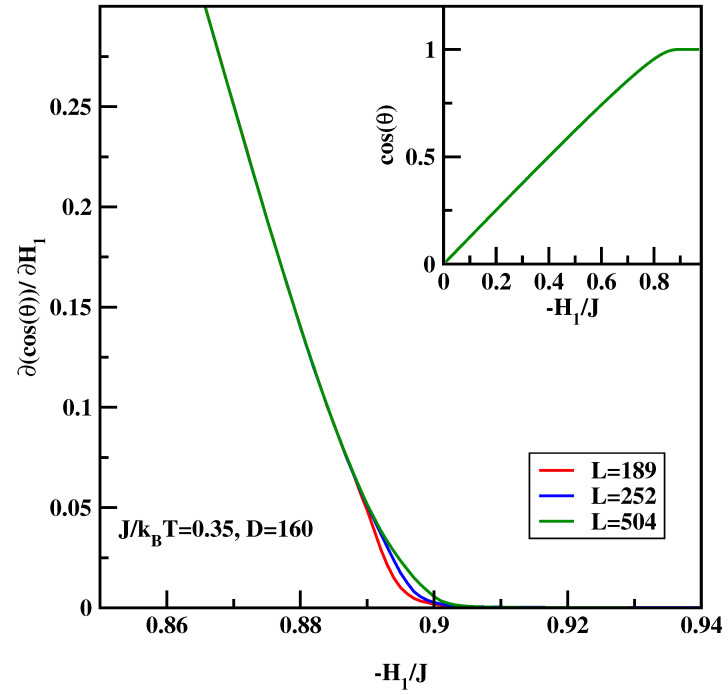
$\partial \cos\left(\theta \right)/\partial H_{1}$ vs. the surface field for $J/k_{B}T=0.35$ and for three system sizes *L* listed in Figure. The inset shows full dependence of cos(θ) vs. $H_{1}/J$.

**Figure 14:**
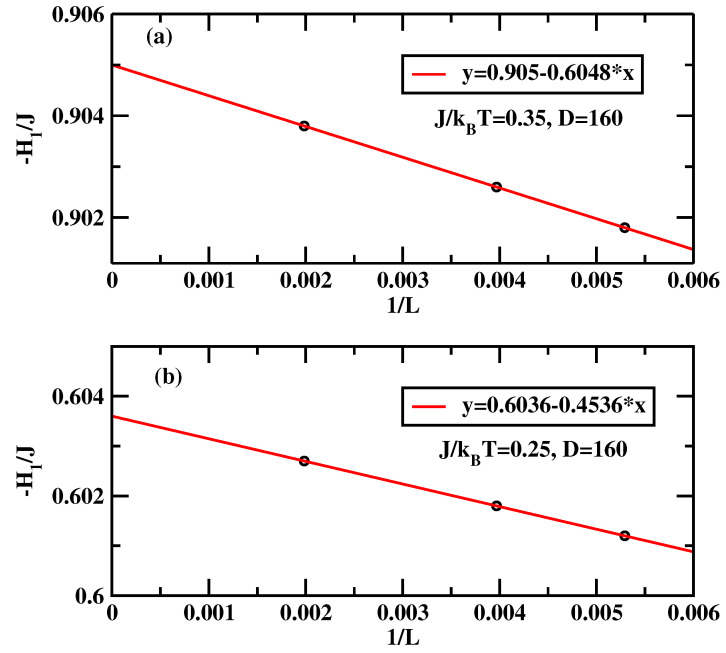
Symbols denote the estimate of the surface field for which $\partial \cos\left(\theta \right)/\partial H_{1}=0$ at a given *L*. Thick lines denote the linear regression extrapolating L→∞. Panel (**a**) is for $J/k_{B}T=0.35$ while panel (**b**) is for $J/k_{B}T=0.25$.

The calculational procedure consists of a series of calculations for the anti-symmetric systems with varying surface field with $\Delta H_{1}=0.001$. We carried out calculations for $J/k_{B}T=0.25$ and 0.35. The integrand of the right-hand-side of Equation (16) depends quite strongly on the system size close to the critical surface field, despite the fact that there is no wetting transition in the system. Therefore we carried out calculations for D=160 and L=189 for surface fields $H_{1}/J=0$ up to $H_{1}/J=-0.511$, while for the stronger surface fields $H_{1}/J<-0.511$ the calculations were carried out for L=189, 252 and 504. Similar to previous calculations hyper-parallel tempering was used with up to 120 systems simulated at the same time. The averages were accumulated over $10^{7}$ spin flips per site. Figure 10 and Figure 11 show the sum of the surface magnetizations divided by the surface tension, i.e., the integrand of Equation (16). We see that close to the critical surface field the integrand develops finite-size dependence. The insets of Figure 12 and Figure 13 show the cosine of the contact angle vs. the surface field. We see that the cosine goes to 1 tangentially. However, it is difficult to pinpoint exactly the tangential point. Moreover, the integral depends on the accuracy of the numerical values of the surface tension. Hence we adopt the following strategy. First we calculated numerically the derivative of the cosine of the contact angle with respect to the surface field (cf. main plots of Figure 12 and Figure 13). The derivative should be zero at the critical surface field, $H_{1c}\left(L\right)$. In order arrive at an estimate of $H_{1c}\left(L\right)$ we plotted two parallel lines $\cos\theta^{\prime }=c_{1}$ and $\cos\theta^{\prime }=c_{2}$ and constructed tangential lines via finite difference. Extrapolation of these straight lines give $H_{1}\left(L\right)$ satisfying $\cos\theta^{\prime }=0$. Finally from the plots of $H_{1}\left(L\right)$ vs. 1/L the thermodynamic limit is reached via extrapolation (cf. Figure 14). The final values of the critical surface fields estimated using this method are $H_{1c}/J=-0.905$ for $J/k_{B}T=0.35$, and $H_{1c}/J=-0.604$ for $J/k_{B}T=0.25$.

### 4.2. BLK Method for Symmetric Surface Fields Revisited

The original idea of Binder, Landau, and coworkers [13,14] (hereinafter referred to as the BLK method) was to consider the symmetric system, $H_{1}=H_{D}$. After setting H=0 the location of the critical wetting transition can be estimated out by varying $H_{1}$ at a constant temperature. The maximum of $\chi_{1}$ as a function of $H_{1}$ indicates the location of the critical surface field $H_{1c}$ at which the critical wetting transition occurs. First simulations were carried out for symmetric field at $J/k_{B}T=0.35$, in order to directly compare with [15]. We assumed that D=60 and L=126, 252, 315, 378, and 504. The statistical effort was $10\times 10^{6}$ spin flips per site (not counting extra gains due to the preferential sampling technique). A total of 16 systems, each at different $H_{1}$ were simulated at once using hyper-parallel tempering technique.

**Figure 15:**
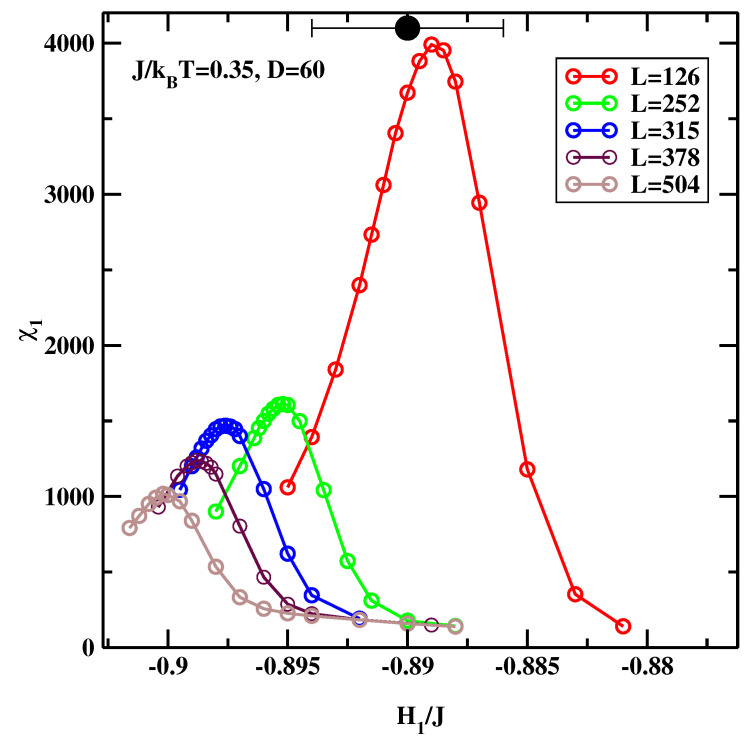
$\chi_{1}$ for five different system sizes *L*, evaluated at $J/k_{B}T=0.35$ for the symmetric surface fields, $H_{1}=H_{D}$. The big black circle with error bars denotes the simulational result of Binder et al. [15].

Figure 15 shows the results of the simulations. Its clear that the statistics is much improved, when compared to the earlier efforts.

**Figure 16:**
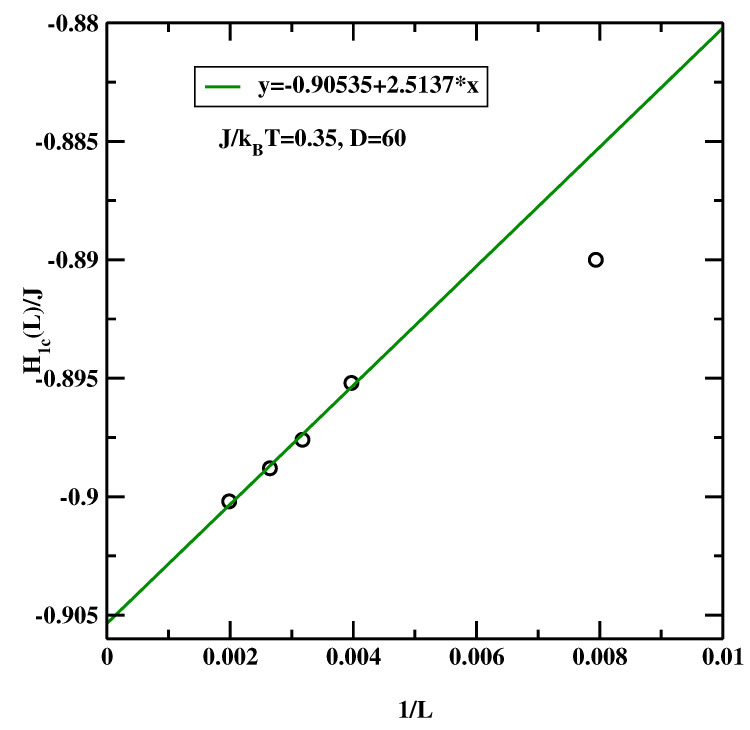
Estimation of $H_{1c}\left(L=\infty \right)$ from simulational data presented in Figure 15, evaluated at $J/k_{B}T=0.35$ for the symmetric surface fields.

The data for L=126 can be compared with that from Ref. [15]. We note that the position of the maximum of $H_{1c}\left(L=126\right)/J=-0.889\pm 0.0005$ agrees very well with earlier study ($H_{1c}\left(L=128\right)/J=-0.89\pm 0.004$). However, there is a considerable shift for larger systems. This indicates that $H_{1c}\left(L=\infty \right)$ is lower. This is confirmed in Figure 16, where we show extrapolation L→∞, and find, that the estimated value $H_{1c}\left(L=\infty \right)/J=-0.905$. In making this extrapolation we did not include data for L=126 due to considerable deviations from the rest of the data.

**Figure 17:**
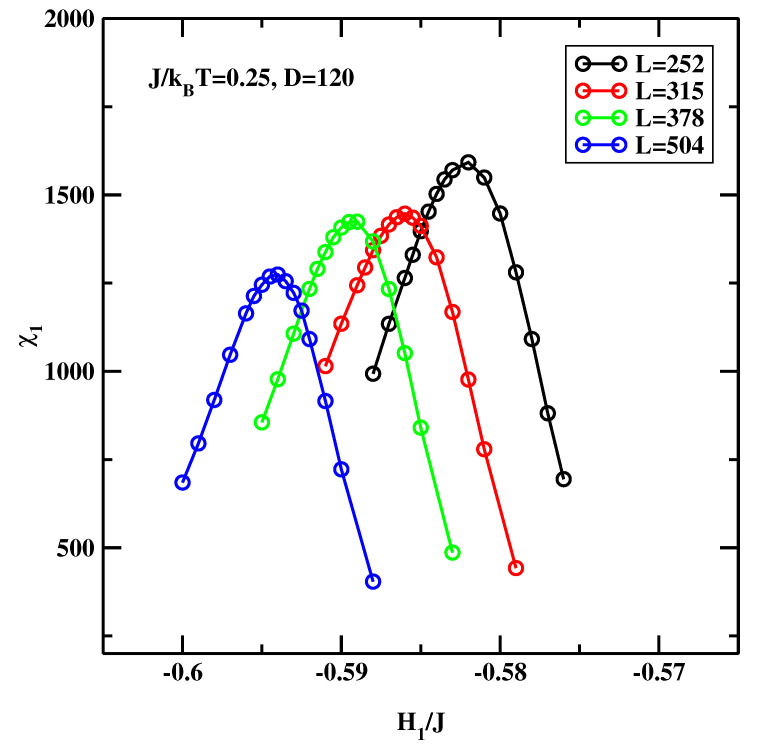
$\chi_{1}$ for different system sizes, evaluated at $J/k_{B}T=0.25$ for the symmetric surface fields.

**Figure 18:**
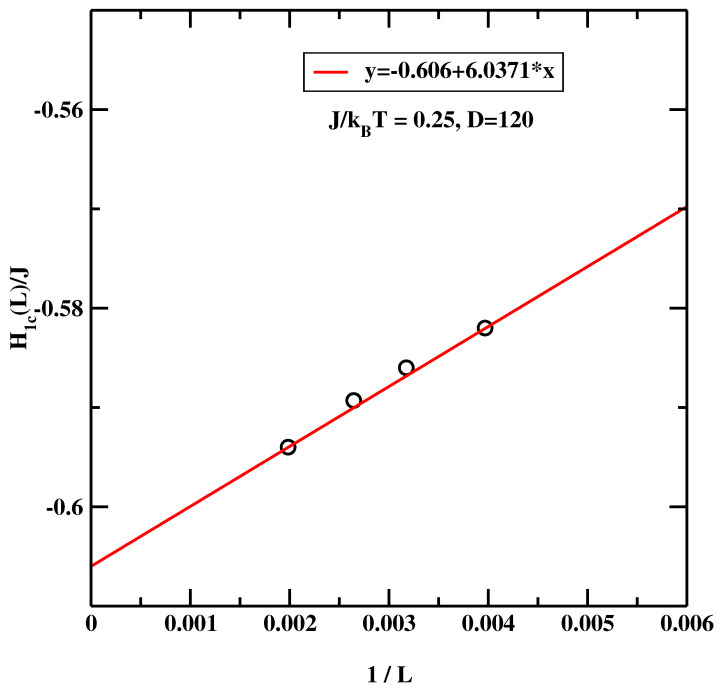
Estimation of $H_{1c}\left(L=\infty \right)$ from simulational data presented in Figure 17, evaluated at $J/k_{B}T=0.25$ for the symmetric surface fields.

Figure 17 shows the results calculated for $J/k_{B}T=0.25$. Since this temperature is closer to the bulk critical temperature the correlation length is greater. For this reason we carried out calculations for somewhat larger systems with D=120 and L=252, 315, 378, and 504. The overall result are similar to those obtained at $J/k_{B}T=0.35$. The extrapolation to L=∞ reveals (cf. Figure 18), that that the estimated value $H_{1c}\left(L=\infty \right)/J=-0.606$. This is again, noticeably lower than previous estimates calculated using much smaller system sizes ($H_{1c}\left(L=\infty \right)/J=-0.555$ [13,14]).

As the careful Reader noticed, there is one vexing feature of the results presented in Figure 15 and Figure 17. Namely the maxima of $\chi_{1}$ get smaller and smaller as the linear system size increases. This means that in the thermodynamic limit this peak disappears and this transition does not exist! We recall that the simulated system is not semi-infinite but forms a slit-like pore. In such systems the only phase transition that remains stable is capillary condensation. It’s clear that the large statistical effort and hyper-parallel tempering technique must give *the correct result*, i.e., that the critical wetting transition studied in this simulational setup is not a stable transition in the thermodynamic limit. However, we argue that in this particular case one can still trust the positions of the maxima of the plots of $\chi_{1}$ even if their magnitudes decrease with increasing system size. This situation can be interpreted in terms of finite size scaling at the first order transition, as formulated by Binder and Landau [35]. One can approximate the probability distribution of the magnetization of an Ising system by two Gaussians for the two phases that would coexist at the capillary condensation transition. At zero bulk field, the phase with the magnetization oppositely oriented to the surface fields is not the stable one. It still gives a signal in a finite system, which ultimately-in the thermodynamic limit - will be exponentially suppressed. However, for the *surface* susceptibility, the stable phase (magnetization parallel to the surface field) gives only a small background contribution. Hence one can still detect the developing singular behaviour of the surface layer susceptibility even though the magnitude of the signal decreases with increasing system size. If the sampling is insufficient to reach full equilibrium, one may get the wrong amplitude of the signal, but it will be still possible to detect the location of the anomaly [36]. To conclude this subsection, the BLK method yields the critical surface fields $H_{1c}\left(L=\infty \right)/J=-0.905$ for $J/k_{B}T=0.35$ and $H_{1c}\left(L=\infty \right)/J=-0.606$ for $J/k_{B}T=0.25$.

## 5. Discussion

The results showed in the previous Sections are summarized in Table 1.

We note that all three methods considered in this work yield results consistent with the AFSS method. At the same time there is a huge difference between the results of the original BLK method and the rest of the results. This is however to be expected, since at the time of carrying out the calculations in [13,15], it was technically impossible to go beyond the system sizes considered in those papers. Each of the presented here methods has some advantages and drawbacks. The TIN method while very promising proves to be tricky due to the lack of proper finite-size analysis. The BLK method requires calculations for progressively large system sizes. The EIP method requires the bulk fluctuations to be small (relative to the capillary wave fluctuations), hence it is not suitable for determination of the critical wetting transition close to the bulk critical point.

In terms of the computational effort the TIN method is least demanding. This is due to the fact that the interfacial fluctuations associated with critical wetting are absent, when using this route. Hence the statistical effort measured as the number of attempted spin flips per site can be two orders of magnitude smaller. The other methods are computationally more involving. The EIP method seems to be slightly less demanding than the BLK method due to the fact that there is no need to consider very large system sizes.

## 6. Conclusions

We have studied three methods of determination of the critical wetting transition. Our findings can be summarized as follows:

- The effective interface potential method can be used to determine the location of the critical wetting transition. The limitation of this method is that its accuracy decreases if the bulk fluctuations become important.
- The thermodynamic integration method can be used to estimate the location of the critical wetting transition. Extrapolation to the thermodynamic limit is non-trivial.
- The Binder–Landau–Kroll method of determination of the critical wetting transition also leads to reasonable results if sufficiently big system sizes are considered.

Recently Evans et al. showed [37] that the Nakanishi and Fisher [9] topology of the global surface phase diagram is not complete. Their study unveils novel classes of the surface phase diagram which are not present for lattice models. We hope that our work will be useful in establishing simulational tools that will help in better understanding of the origin of the differences between the atomistic and lattice models. In our opinion the results of the wetting behaviour for the simple lattice models have to be revisited as well, since the old estimates were computed for small system sizes. Some of these issues are being currently considered.

## Figures and Tables

**Table 1:** Critical surface field $H_{1c}/J$ obtained from various methods.

$J/k_{B}T$	AFSS ([18])	EIP (This Work)	TIN (This Work)	BLK (This Work)	BLK ([13,15])
0.25	−0.616	−0.608	−0.604	−0.606	−0.555
0.35	−0.909	−0.908	−0.905	−0.905	−0.89

## Data Availability

Data used in this research can be obtained from the Authors upon a reasonable request.
